# Nectar Sugar Modulation and Cell Wall Invertases in the Nectaries of Day- and Night- Flowering *Nicotiana*

**DOI:** 10.3389/fpls.2018.00622

**Published:** 2018-05-09

**Authors:** Kira Tiedge, Gertrud Lohaus

**Affiliations:** Molecular Plant Science/Plant Biochemistry, University of Wuppertal, Wuppertal, Germany

**Keywords:** floral nectar, nectaries, cell wall invertase, tobacco, *Nicotiana*, sugar composition, diurnal/nocturnal

## Abstract

Nectar composition varies between species, depending on flowering time and pollinator type, among others. Various models of the biochemical and molecular mechanisms underlying nectar production and secretion have been proposed. To gain insights into these mechanisms, day- and night-flowering tobacco (*Nicotiana*) species with high or low proportions of hexoses in the nectar were analyzed. Nectar and nectaries were simultaneously collected, throughout the day and night. Soluble sugars and starch were determined and the activity and expression level of cell wall invertase (CW-INVs) were measured in nectaries. Nectaries and nectar of the five *Nicotiana* species contained different amounts of sucrose, glucose, and fructose. CW-INV activity was detected in the nectaries of all *Nicotiana* species and is probably involved in the hydrolysis of sucrose in the nectary tissue and during nectar secretion. The larger differences in the sucrose-to-hexose-ratio between nectaries and nectar in diurnal species compared to nocturnal species can be explained by higher sucrose cleavage within the nectaries in night-flowering species, and during secretion in day-flowering species. However, cell wall invertase alone cannot be responsible for the differences in sugar concentrations. Within the nectaries of the *Nicotiana* species, a portion of the sugars is transiently stored as starch. In general, night-flowering species showed higher starch contents in the nectaries compared to day-flowering species. Moreover, in night flowering species, the starch content decreased during the first half of the dark period, when nectar production peaks. The sucrose concentrations in the cytoplasm of nectarial cells were extrapolated from nectary sucrose contents. In day-flowering species, the sucrose concentration in the nectary cytoplasm was about twice as high as in nectar, whereas in night-flowering species the situation was the opposite, which implies different secretion mechanisms. The secreted nectar sugars remained stable for the complete flower opening period, which indicates that post-secretory modification is unlikely. On the basis of these results, we present an adapted model of the mechanisms underlying the secretion of nectar sugars in day- and night-flowering *Nicotiana*.

## Introduction

Nectar is a sugar-rich solution which is produced by most angiosperm plants to fulfill extensive functions, e.g., the attraction of pollinators and protection against herbivores (Brandenburg et al., 2009; González-Teuber and Heil, 2009; Adler et al., 2012). Nectar is produced by and secreted from nectaries, which are highly specialized glands, and the surrounding tissue. In *Nicotiana*, all floral nectaries are located at the basal side of the gynoecium (Bernardello, 2007) and during nectary development, β-carotene is expressed, which results in an orange coloring (Horner et al., 2007). The synthesis and secretion of floral nectar has been the subject of several studies, and different models on the biochemical and molecular mechanisms underlying nectar secretion have been proposed (Ge et al., 2000; Horner et al., 2007; Kram et al., 2009; Mosti et al., 2013; Stpiczyńska et al., 2014). But due to the enormous diversity of flowering plants, there are still several variables that warrant further study (Roy et al., 2017). A very basic theory of nectar secretion proposes an apoplastic movement of metabolites from the phloem to the nectary surface (Vassilyev, 2010). However, the metabolite composition differs between the phloem sap and the nectar (Lohaus and Schwerdtfeger, 2014), which does not support the proposed apoplastic method of nectar secretion. Other hypotheses propose that various enzymes and transport proteins are involved in nectar production. For certain plant species, an eccrine secretion mode has been proposed, wherein sucrose is delivered from the phloem to the nectary parenchyma cells, and there the sucrose is transiently converted to starch or exported to the apoplast directly. A plasma membrane-localized sucrose transporter SWEET9 is essential for this transport (Lin et al., 2014). SWEET9 functions as a facilitated diffusion transporter for sucrose, and mutants lacking SWEET9 do not produce nectar, e.g., in *Nicotiana attenuata* (Lin et al., 2014). Once sucrose is exported from the nectary, it is then hydrolysed by an extracellular cell wall invertase (CW-INV) into glucose and fructose (Ruhlmann et al., 2010). In a third proposed secretory mechanism, nectar metabolites are transported symplastically to the outer nectary cells and then packed into vesicles, which are produced by the endoplasmic reticulum (ER) or the Golgi complex, to fuse with the plasma membrane and release the nectar metabolites to the nectary surface (Fahn, 1979a,b). These three models for nectar secretion are not necessarily mutually exclusive, and other modes of nectar secretion can occur in different plant species.

In some plant species, starch accumulates in the nectaries and peaks approximately 24 h before anthesis and then declines rapidly, which is the basis for the hypothesis that starch is one source of sugars for nectar production before and during nectar secretion (Nepi et al., 1996; Horner et al., 2007; Ren et al., 2007a,b). Genes encoding anabolic enzymes involved in starch synthesis were found to be more highly expressed at the early stages of nectary development, and genes encoding catabolic enzymes were expressed at later stages (Ren et al., 2007a). However, studies on lychee (*Litchi chinensis*) floral nectaries have shown that the nectar sugar is composed of both phloem sap and products of starch degradation in the nectaries (Ning et al., 2017).

Of the sugars found in nectar, the most prevalent are sucrose and the hexoses glucose and fructose (Percival, 1961; Baker and Baker, 1983; Tiedge and Lohaus, 2017). Given that hexoses are typically not components of the phloem sap (Lohaus and Schwerdtfeger, 2014), the proportion of hexoses in nectar depends on the presence and activity of sucrose-cleaving enzymes. Sucrose cleavage in plants can be catalyzed by at least two types of enzymes: reversible sucrose cleavage is catalyzed by sucrose synthase (SuS; EC 2.4.1.13), a glycosyltransferase; and irreversible sucrose cleavage is catalyzed by invertases, which catalyze hydrolysis (β-fructofuranosidases; EC 3.2.1.26). Invertases exist in numerous isoforms with various subcellular localizations and biochemical properties (Roitsch and González, 2004). These enzymes can be classified into three groups: vacuolar invertases (V-INVs), extracellular invertases (CW-INVs), and neutral invertases (N-INVs). Whereas N-INVs have an alkaline pH-optimum, V-INVs and CW-INVs are so-called “acidic invertases” because they work most efficiently between pH 4.5 and 5.0. Extracellular invertases are non-soluble proteins that are ionically bound to the cell wall (Sturm, 1999). A separate gene encodes for each of the isoforms, which have a high identity and share common features, e.g., the pentapeptide NDPNG (βF-motif) close to the N-terminus of the mature protein, and WECXDF, an amino acid sequence closer to the C-terminus (Sturm and Chrispeels, 1990; Roitsch and González, 2004).

For some plant species, e.g., carrot (*Daucus carota*) and tomato (*Solanum lycopersicum*), different organ- and development-stage-specific expression patterns of acid invertase were shown (Sturm et al., 1995; Godt and Roitsch, 1997). Usually, invertase expression is increased in rapidly growing tissues with a high demand for hexoses (Weschke et al., 2003). Interestingly, for both carrot and tomato, the mRNA expression of an acidic invertase was found to be specific to flowers and flower buds (Lorenz et al., 1995; Godt and Roitsch, 1997). It was assumed that this flower-specific extracellular invertase is essential for male and female organ development, e.g., to supply the anthers with carbohydrates (Dorion et al., 1996; Godt and Roitsch, 1997). More recently, it was shown that CW-INV is also crucial for nectar secretion in *Arabidopsis* (Ruhlmann et al., 2010). AtCWINV4 expression was found to be highly up regulated in nectaries of *A. thaliana* compared to other tissues (Kram et al., 2009). Furthermore, two independent *cwinv4*-mutant lines with greatly diminished activity of total CW-INV in whole *Arabidopsis* flowers secreted no nectar, although the nectary ultrastructure appeared to be similar to that of wild-type plants (Ruhlmann et al., 2010).

The genus *Nicotiana* is highly diverse in terms of flower morphology and pollination mode. In a study involving 20 *Nicotiana* species, the sugar concentration in the nectar of several day- and night flowering species was measured (Tiedge and Lohaus, 2017). The genus *Nicotiana* contains species with sucrose-rich nectars as well as hexose-rich nectars, and the exact nectar composition depends on the pollinator type, flowering time, corolla length and other environmental factors (Tiedge and Lohaus, 2017). The sucrose-to-hexose ratio ranged from 0.1 to 2.0 and was fairly consistent within a given species.

This finding raises the question of whether the sugar composition in nectar is a result of the sugar composition in the nectaries. Alternatively, a lower sucrose content in nectar could reflect higher invertase activity in the nectaries and during nectar secretion. Furthermore, we aimed to investigate potential differences in invertase expression and activity over the course of a day, in consideration of flower opening and nectar production times. In addition to these pre-secretory and secretory processes, post-secretory processes could also be responsible for varying sugar composition. In such a scenario, the nectar itself must contain sugar cleaving enzymes.

**FIGURE 1:**
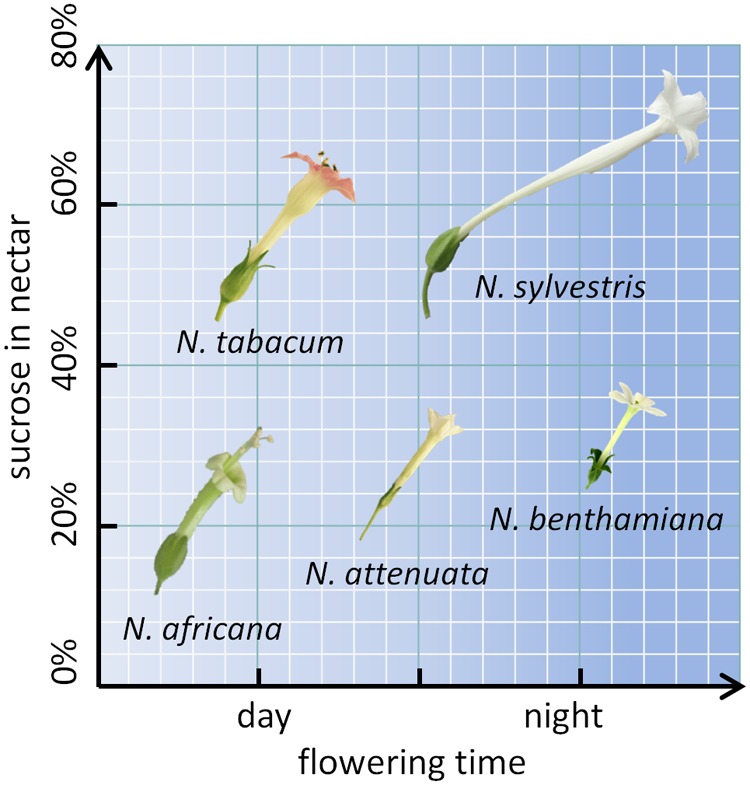
*Nicotiana* species arranged by flowering time and sucrose content. The flower sizes are depicted to relative scale.

To further investigate the mechanism underlying nectar production and secretion, five tobacco species with varying properties were examined. Two day-flowering species (*N. tabacum* and *N. africana*) as well as two night-flowering species (*N. sylvestris* and *N. benthamiana*) were included. Within each category (day- or night-flowering), one species had a high sucrose content and one species had a low sucrose content (**Figure 1**). For reproduction, these species rely on pollination either by diurnal birds (*N. africana*: sunbirds; *N. tabacum*: hummingbirds), nocturnal moths (*N. sylvestris*), or otherwise the species is primarily autogamous (*N. benthamiana*) (Tiedge and Lohaus, 2017). Additionally, *N. attenuata* was chosen, which opens its flowers at twilight both in the evening and in the morning and is therefore less dependent on a specific pollinator (Kessler and Baldwin, 2007). To investigate whether the nectar sugar content primarily depends on pre-secretory processes, the secretion process, or post-secretional modification, nectar sugars were compared to nectary sugars at multiple time points per day; additionally, the invertase activity and expression were measured, and post-secretional activity was recorded.

## Materials and Methods

### Plant Material

*Nicotiana attenuata* seeds were provided by the Max Planck Institute for Chemical Ecology (Jena, Germany), *N. benthamiana* seeds were provided by the University of Rostock (Germany), *N. africana* and *N. sylvestris* seeds were provided by the Botanical Garden of Ruhr University Bochum (Germany), and *N. tabacum* seeds were provided by NiCoTa (Rheinstetten, Germany). Each plant was potted in a single 5-L pot with compost soil and grown in a greenhouse at the University of Wuppertal. Cultivation was carried out with a 14-h-light/10-h-dark cycle, an irradiance of approximately 300 μmol photons m^-2^ s^-1^ and a temperature regime of 25°C day/18°C night.

### Collection of Nectaries and Nectar

Each sample (∼100 mg) of nectary tissue comprised 20–50 nectaries, depending on the species. At each time point (2 p.m., 8 p.m., 2 a.m., and 8 a.m.), three samples were taken. To collect the nectaries, the gynoecia were extracted from the flowers, and the nectary tissue was dissected with a scalpel and rinsed with ultrapure water to remove external sugars. All samples were immediately frozen in liquid nitrogen and stored at -80°C until further analysis. The weight of a single nectary was calculated as follows:

Weight per floral nectary [mg] = 100mg/number of nectaries collected per sample

For each species, at least three nectar samples were taken from three plants at all four time points. The nectar samples were collected with micropipettes, assayed for microbial contamination according to Tiedge and Lohaus (2017) and stored at -80°C until further analysis. In addition, nectar samples were also analyzed by light-microscopy to exclude contamination with pollen. The nectar samples used for post-secretional experiments were left at room temperature for 12, 24, and 48 h. The water content of the nectaries and leaves was determined by drying and weighing those tissues. The following calculation was used:

Water content = 1−(dry weight [mg]/fresh weight [mg])

### Analysis of Sugars and Starch in Nectaries and Nectar

For the extraction of soluble metabolites from nectary tissue, a chloroform-methanol-water extraction was performed (Nadwodnik and Lohaus, 2008). The analysis of sugars in nectar, nectaries and leaves via HPLC was conducted according to Lohaus and Schwerdtfeger (2014). Nectar was filtered (0.2 μm nitrocellulose; Schleicher and Schuell, Germany) before HPLC measurements to exclude contamination with pollen. An ion exchange column (CarbopacTM PA10 4 mm × 250mm; Dionex Corp, Sunnyvale, CA, United States) was eluted isocratically with 80 mM NaOH (JT Baker Chemicals). Sugars were detected with a pulse amperometric detector with a gold electrode (ESA Model 5200, Coulochem II, Bedford, MA, United States). The pulse setting was 50, 700, and -800 mV for 400, 540 and 540 ms, accordingly. For external calibration, sugar standards (Sigma-Aldrich, Germany) were measured in parallel. The evaluation of the chromatograms was performed with an integration program (Peaknet version 5.1, Dionex). Starch content of nectaries was determined according to a modified protocol from Riens et al. (1994).

### Expression of CWINV

RNA from approximately 50 mg of nectariferous tissue was isolated using a modified protocol from Logemann et al. (1987), where cetyltrimethylammonium bromide (CTAB) is used to inactivate RNase activity and to form a complex with RNA without adding guanidine. Synthesis of cDNA was performed using the RevertAid^TM^ First Strand cDNA Synthesis Kit (Thermo Fisher Scientific, Sankt Leon-Rot, Germany) with oligo(dT)_18_ primers. Degenerated primers were designed to amplify CW-INV sequences of the different *Nicotiana* species. The obtained sequences were cloned with the pGEM^®^-T Easy Vector System (Promega Corporation, Madison, IW, United States) for sequencing, and suitable specific primers for quantitative real-time polymerase-chain-reaction (qRT-PCR) were selected. For verification of the obtained primers and sequences, amplification with proof read polymerase (Phusion High-Fidelity DNA-Polymerase, Thermo Fisher Scientific, Waltham, MA, United States) and blasting with known sequences from NCBI (National Center for Biotechnology Information, Bethesda, MD, United States) was performed. QRT-PCR analyses were performed using a Maxima SYBR Green qPCR Master Mix (Thermo Fisher Scientific, Waltham, MA, United States) and a Mx3005P qPCR System (Agilent Technologies Inc., Waldbronn, Germany). Efficiencies of the PCRs were calculated with slopes of standard curves of twofold dilutions. For each species two stable reference genes were used for normalization (Vandesompele et al., 2002; Schmidt and Delaney, 2010; Liu et al., 2012). The first sample of each experiment was used as a calibrator, which was set to one, and further samples are given as relative expression levels to the calibrator. For each condition three biological replicates with two technical replicates each were tested. A list of the primers used for each species can be found in Supplementary Table 1.

### Enzyme Assay for CWINV, Soluble Acid Invertase, and Neutral Invertase

Proteins were extracted from 25 mg nectary tissue each as described by Wright et al. (1998). CW-INV activity was assayed according to Heineke et al. (1992). An aliquot of the protein extracts was added to 0.6 M sucrose and 0.125 M sodium acetate, pH 5.0. Soluble acid invertase activity was measured with the soluble protein fraction. An aliquot of the protein extracts was added to 0.6 M sucrose and 0.125 M sodium acetate, pH 5.0. Soluble neutral invertase activity was measured with the soluble protein fraction, too. An aliquot of the protein extracts was added to 0.6 M sucrose and 0.125 M sodium acetate, pH 7.5. After 10 min, the reaction was completely stopped by boiling and subsequently, the amount of glucose released was determined by coupled optical enzyme assay. All enzyme assays were conducted from six biological replicates with two technical replicates each. About 5 μL of nectar were also used to assay invertase activity.

## Results

### Sugar Concentrations in Nectar and Nectaries During the Light and Dark Period

**FIGURE 2:**
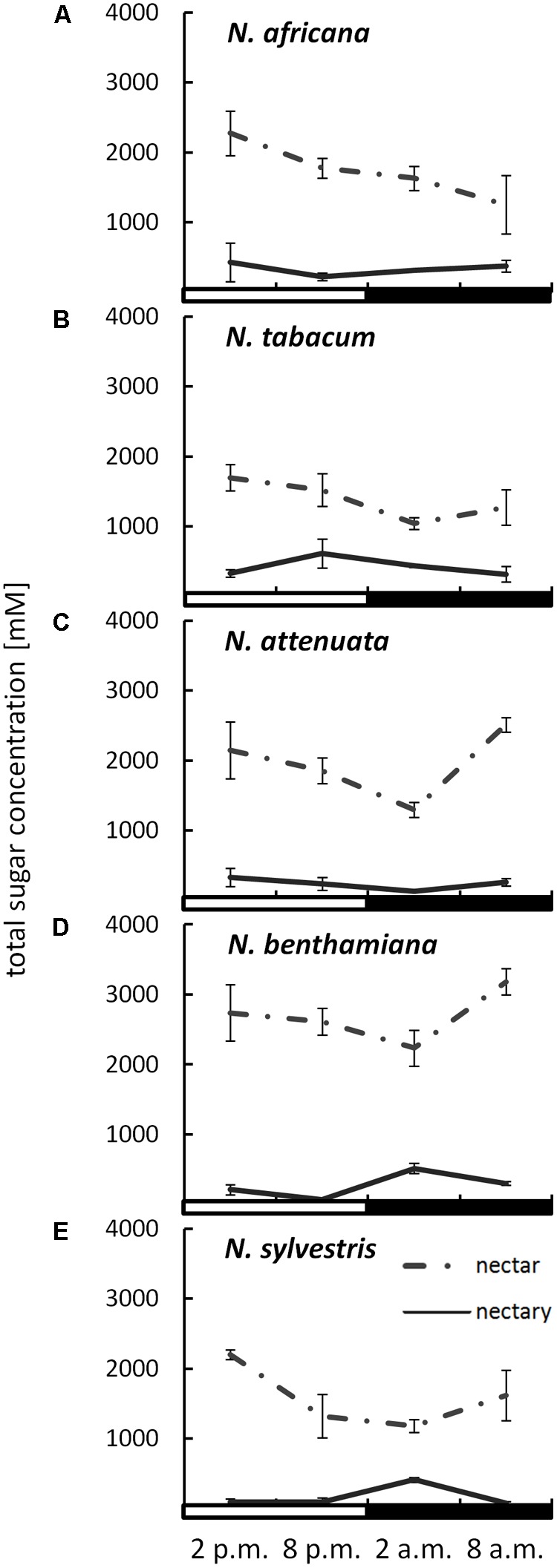
Sugar concentrations in nectaries and nectar. Mean values from all measurements taken at one time point (*n* = 3) and the respective SDs were plotted; light and dark periods are indicated by white and black bars. **(A)** *N. africana*, **(B)** *N. tabacum*, **(C)** *N. attenuata*, **(D)** *N. benthamiana*, and **(E)** *N. sylvestris*.

The sugar content in both nectar and nectaries was primarily composed of glucose, fructose, and sucrose. Other sugars, including maltose, were not found in any of the samples. The total sugar concentration in nectar ranged from 1042 ± 86 to 3183 ± 186 mM, depending on the species and collection time (**Figure 2**). The day-flowering species (*N. africana* and *N. tabacum*) had the highest nectar sugar concentration during the day, which decreased continuously at night. In the case of night-flowering tobacco (*N. benthamiana, N. sylvestris*), the lowest sugar concentration in nectar was also found in the first half of the night period, but the concentration increased during the second half of the night period. Day- and night-flowering *N. attenuata* behaved like *N. benthamiana* (**Figure 2**).

By measuring the sugar content in the nectaries in micromole per gram fresh weight and the water content of the nectaries, it was also possible to determine the sugar concentration in the nectaries. The total sugar concentration in the nectaries of all species was lower than in the nectar, ranging from 72 ± 6 to 613 ± 34 mM (**Figure 2**). The mean sugar concentration in the nectar was approximately three to fivefold higher than in the nectaries of day flowering species, and approximately eight to 10-fold higher in night flowering species and in *N. attenuata*. In the day-flowering plants, the highest sugar concentration in nectaries occurred either in the middle or at the end of the light period (2 or 8 p.m.). The same phenomenon applied to the mixed-type *N. attenuata*. In both night-flowering plants, the sugar concentration in nectaries increased sharply in the middle of the night at 2 a.m.

The leaves of these tobacco species also contained primarily sucrose, glucose, and fructose. Independent of the flowering time, the sugar content in leaves was higher at the end of the light period than at the end of the dark period (Supplementary Figure 1). When compared to nectaries or nectar, leaves had a significantly lower sugar concentration (10–60 mM). These results were derived from the sugar content per gram fresh weight and the corresponding water content (78–94%; data not shown).

Nectar samples have been tested for microbial contamination. However, no contaminations with yeast or bacteria in the different *Nicotiana* species were found and therefore externally induced changes in the nectar sugar profile due to microbial activity can be excluded.

### Sugar Composition in Nectar and Nectaries During the Light and Dark Period

**FIGURE 3:**
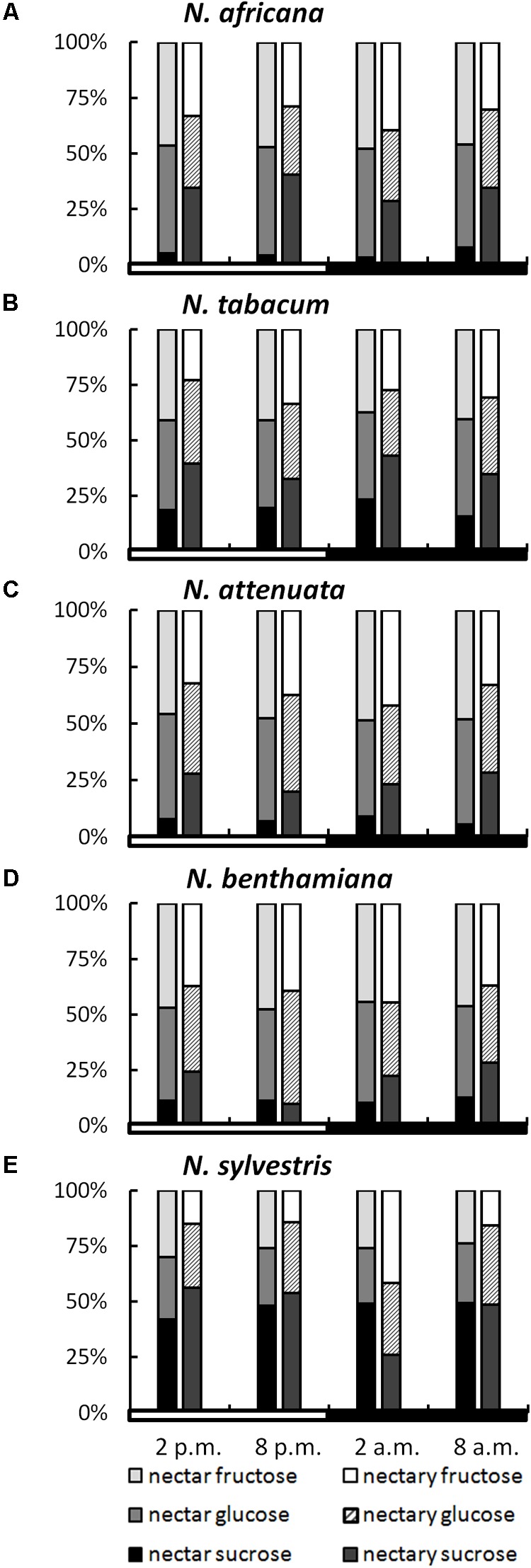
Sugar percentages in nectaries and nectar. All percentages were calculated from mM; *n* = 3; light and dark periods are indicated by white and black bars; one pair of bars indicates one time point of sampling, with the Left bar representing nectar and the Right bar representing nectaries. **(A)** *N. africana*, **(B)** *N. tabacum*, **(C)** *N. attenuata*, **(D)** *N. benthamiana*, and **(E)** *N. sylvestris*.

While the ratios of the three sugars within a species remained relatively constant, even during different collection times, the sugar ratio between species varied greatly in some cases (**Figure 3**). This phenomenon was observed for both nectar and nectaries. In the nectar of *N. africana*, the percentage of sucrose ranged from 3–8%, depending on the time of day. Other species with a low sucrose-to-hexoses ratio in nectar were *N. attenuata* and *N. benthamiana*, for which the proportion of sucrose ranged from 6–9 and 10–13%, respectively. Higher proportions of sucrose were found in *N. tabacum* and *N. sylvestris* (16–23 and 42–49%). In general, glucose and fructose were found to occur in similar proportions within a species.

In nectaries, the distribution of sugars was also similar within a species during the light and dark period. In relation to nectar, the percentage of sucrose was higher in nectaries of all *Nicotiana* species, with the exception of *N. sylvestris* at 2 a.m. The percentage of sucrose was relatively low in *N. attenuata* and *N. benthamiana* (10–28%), medium in *N. africana* and *N. tabacum* (26–43%), and high in *N. sylvestris* (26–56%).

To assess whether a percentage increase of a given sugar in nectar was also reflected in the nectaries, the sugar content in both compartments was correlated. For glucose, no bivariate correlation was found (Pearson’s *r* = 0.191, *p* = 0.420), whereas the percentage of both fructose and sucrose between the nectar and nectaries was correlated either highly significantly or significantly (fructose: Pearson’s *r* = 0.574, *p* = 0.008^∗∗^; sucrose: Pearson’s *r* = 0.481, *p* = 0.032^∗^).

However, in all species, the mean sucrose-to-hexoses ratio was higher in nectaries compared with nectar (**Table 1**). In general, the difference between the sucrose-to-hexoses ratios in nectaries and nectar was higher in light flowering species (Δ 0.37–Δ 0.48) compared with night flowering species (Δ 0.05–Δ 0.15).

**Table 1:** Nectary- and Nectar-sugar-ratios (calc. from mM) Data were derived from **Figures 2, 3**, the values of all measuring points were averaged.

Species	Nectary-sugar-ratio [S/(G+F)]	Nectar-sugar-ratio [S/(G+F)]	Difference between the ratios
*N. africana*	0.54	0.05	0.48
*N. tabacum*	0.61	0.24	0.37
*N. attenuata*	0.33	0.08	0.25
*N. benthamiana*	0.28	0.13	0.15
*N. sylvestris*	0.94	0.89	0.05

### Starch Content in Nectaries

**FIGURE 4:**
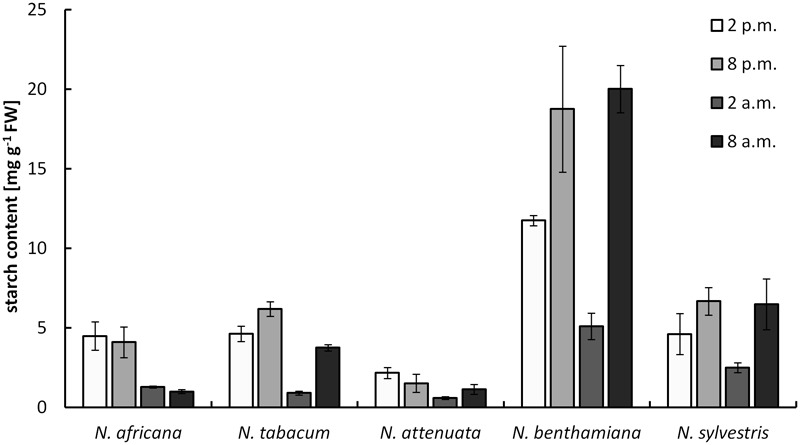
Starch concentration in nectaries. Mean values from all measurements taken at one time point (*n* = 3) and the respective SDs were plotted.

The starch content measured in nectaries ranged from 0.9 ± 0.1 mg g^-1^ FW up to 20 ± 1.5 mg g^-1^ FW (measured as glucose equivalent; **Figure 4**). The values were significantly higher in night- than in day-flowering species (*p* = 0.025). The lowest starch contents during the light and dark period were found in the day-flowering species, as well as in *N. attenuata*. Moreover, in these species, the starch content was lower during the dark period and higher during the light period. In the night-flowering *Nicotiana* species, the highest starch contents were found both in the morning and in the evening (**Figure 4**). At 2 a.m., the night flowering species showed the lowest starch levels; thus, at the same time, the night-flowering species presented the highest sugar concentration. Because starch in plants is synthesized from glucose, it has been tested whether there is a correlation between the glucose and starch content in the nectaries, but no significant correlation was found between glucose and starch content or between fructose or sucrose and starch content.

Starch content in leaves ranged from 0.5 ± 0.1 to 40 ± 4.8 mg g^-1^ FW (measured as glucose equivalent; Supplementary Figure 2). For all five species, the starch content in the leaves was higher at the end of the light period compared with the end of the dark period (Supplementary Figure 2). The starch content of nectaries and leaves was not correlated.

### Invertase Activity in Nectaries

**FIGURE 5:**
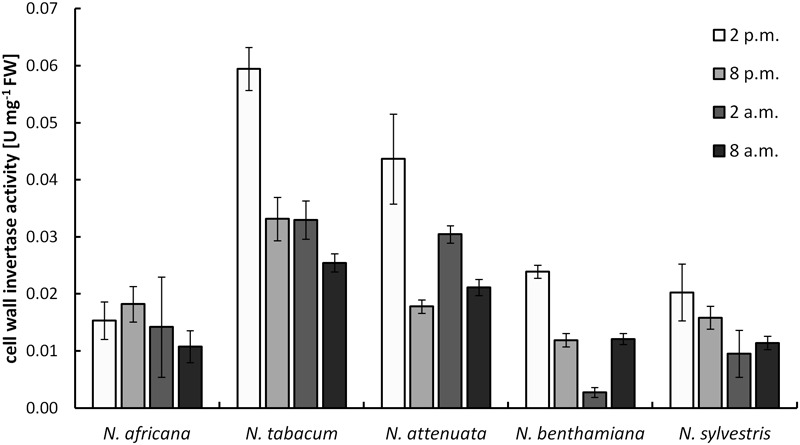
Cell wall invertase (CW-INV) activity in nectary tissue. Mean values from all measurements taken at one time point (*n* = 3) and the respective SD were plotted.

Cell wall invertases in nectaries were active during the light as well as during the dark period. Measured activity ranged from 0.003 ± 0.001 to 0.059 ± 0.004 U mg^-1^ FW (**Figure 5**). Except for *N. africana*, the highest activity levels in all species were found at the middle of the light period, and then the activity decreased, regardless of when the plant opens its flowers. The activity of CW-INV in nectaries did not correlate with any of the sugars in the nectar or the nectaries.

The CW-INV activity in the leaves ranged from 0.003 ± 0.001 to 0.033 ± 0.004 U mg^-1^ FW (Supplementary Figure 3). Therefore, the activity levels were similar to those in the nectaries. CW-INV activity in the leaves fluctuated only slightly between the light and dark periods.

Soluble acid invertases in nectaries were also active during the light as well as during the dark period, but the mean activity was about threefold lower when compared to the CW-INV activity. Measured activity ranged from 0.003 ± 0.001 to 0.013 ± 0.007 U mg^-1^ FW (Supplementary Figure 4A). In the day-flowering species and in *N. attenuata*, the highest activity levels were found at the middle of the light period, whereas in the night-flowering species the highest activity levels were found at the middle of the dark period.

The activity of the soluble neutral invertase in the nectaries of the different *Nicotiana* species was very low (Supplementary Figure 4B). Measured activity ranged from 0.001 to 0.007 U mg^-1^ FW and no significant differences between the species or the sampling points were found.

### Expression Levels of CWINV

**FIGURE 6:**
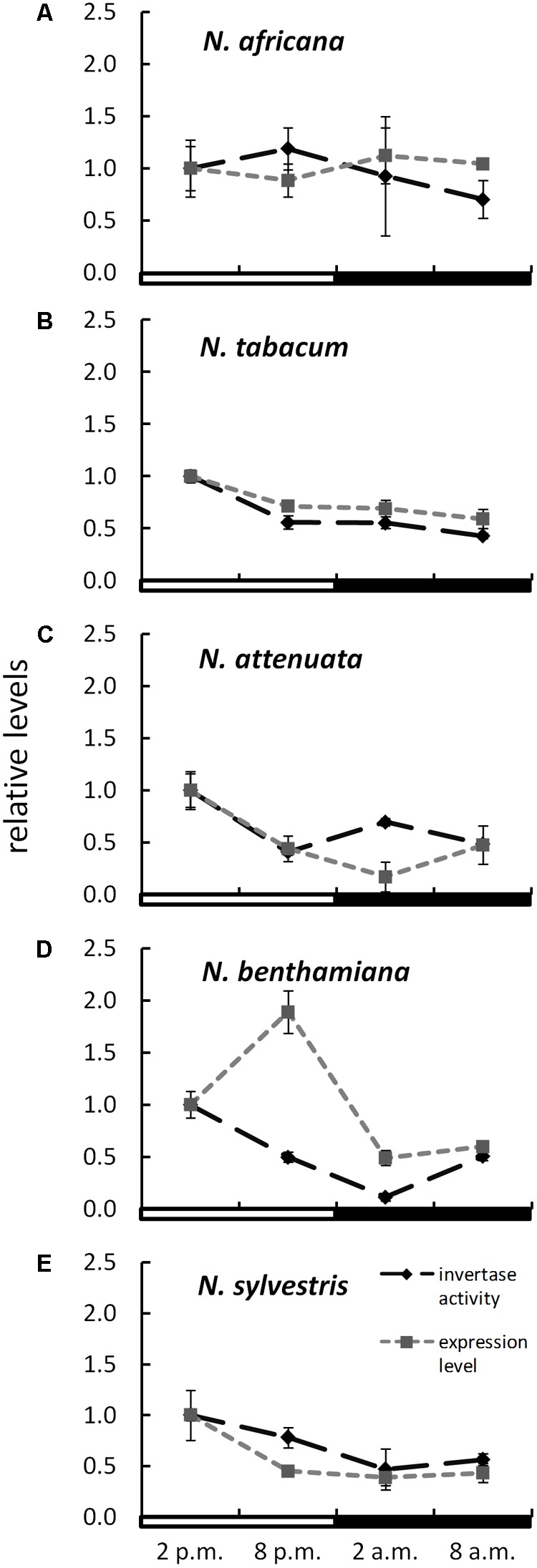
Invertase activity in comparison to expression level in the nectaries of different *Nicotiana* species Activity and expression levels are given relative to the calibrator (2 p.m.). Mean values from all measurements taken at one time point (*n* = 6 for enzyme activity and *n* = 3 for expression levels) and the respective SD were plotted; light and dark periods are indicated by white and black bars. **(A)** *N. africana*, **(B)** *N. tabacum*, **(C)** *N. attenuata*, **(D)** *N. benthamiana*, and **(E)** *N. sylvestris*.

The expression level of CW-INVs in the nectaries of the five *Nicotiana* species was also measured. Therefore, the expressed sequence tag (EST) of the CW-INV of each species was cloned. Specific primers were designed and used for quantitative RT-PCR. In the day-flowering and hexose-rich species, *N. africana*, the relative expression of CW-INV was very stable throughout the light and dark periods. In the other *Nicotiana* species, the expression level was slightly higher during the light period compared to the dark period, regardless of flowing time or the percentage of hexoses in the nectar (**Figure 6**). In most *Nicotiana* species, the course of the expression level was consistent with the invertase activity, especially for *N. tabacum* and *N. sylvestris* (**Figures 6B,E**), but less for *N. benthamiana* (**Figure 6D**). A comparison of the invertase expression level with the nectary sugar concentration revealed a non-homogeneous pattern: In *N. attenuata*, nectary sugars correlated strongly but not significantly with expression level (glucose: Pearson’s *r* = 0.913, *p* = 0.458; fructose: Pearson’s *r* = 0.917, *p* = 0.456; sucrose: Pearson’s *r* = 0.917, *p* = 0.455), whereas in *N. benthamiana*, this correlation was strongly negative (glucose: Pearson’s *r* = -0.887, *p* = 0.469; fructose: Pearson’s *r* = -0.822, *p* = 0.497; sucrose: Pearson’s *r* = -0.963, *p* = 0.437). For the remaining species, the correlation was generally lower.

### Post-secretional Nectar Changes

**FIGURE 7:**
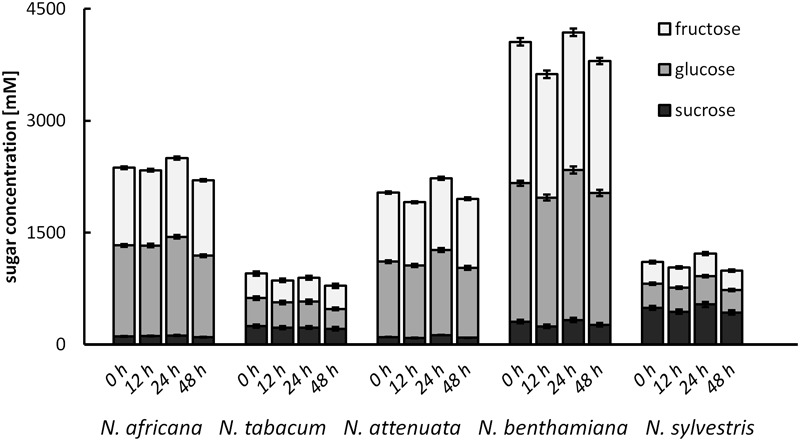
Post-secretional changes in sugar content in the nectar All samples were analyzed immediately after collection, as well as 12, 24, and 48 h after collection; *n =* 3.

To test for changes of the nectar sugar composition after secretion, nectar of all species was measured immediately after sampling, as well as 12, 24, and 48 h later. The results showed that the sugar concentrations were not changed significantly during this period (**Figure 7**). Minor fluctuations were likely caused by the high dilution factor (1: 2000) used to measure nectar with the HPLC. No invertase activity was found in any nectar sample.

## Discussion

Floral nectar is synthesized and secreted by different types of floral nectaries. Nectar composition varies between species, possibly to reward different types of pollinators. Until now, the plant-specific differences in nectar production and nectar secretion that lead to different nectar composition have not been fully understood.

### Pre-secretory Modifications of Nectar Sugars

The phloem supplies the nectaries with sucrose (Lohaus and Schwerdtfeger, 2014). In contrast to phloem sap, where no hexoses are found (Knop et al., 2001; Nadwodnik and Lohaus, 2008), the nectar of the *Nicotiana* species contains substantial amounts of glucose and fructose, in addition to sucrose. Differences in the composition of nectar and phloem may be due to either metabolic processes in the nectaries during nectar secretion or post-secretional modification. To clarify this question, the sugar composition of the nectar and nectaries was compared.

**FIGURE 8:**
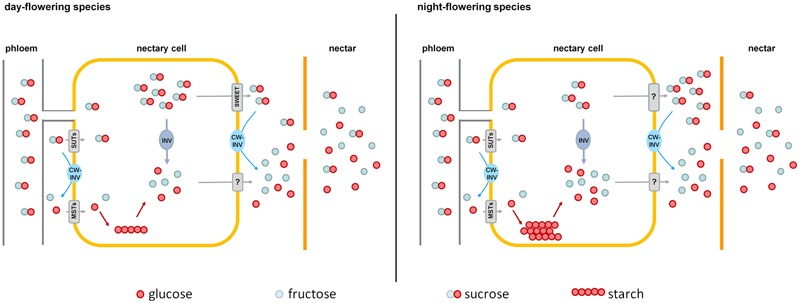
Comparison of nectar sugar secretion in day- and night-flowering tobacco. Day-flowering species **(Left)** store less starch in their nectaries and contain more sucrose in the cytoplasm, which could be exported by SWEET-proteins along the concentration gradient. Night-flowering species store more starch in their nectaries and sucrose probably cannot be exported by SWEETs, since the concentration of sucrose in the cytoplasm is lower than in the nectar. In night-flowering species, sucrose cleavage within the nectaries accounts for a higher proportion of the hexose provision than cleavage during secretion, which is the opposite in day-flowering species. MSTs, monosaccharide transporters; SUTs, sucrose transporters; CW-INV, cell wall invertases; INV, invertase; SWEETs, sucrose efflux transporters; ?, unknown processes.

In the case of night-flowering tobacco, the lowest sugar concentration in nectar was observed in the first half of the dark period (**Figure 2**). This could be due to the fact that the nectar volume in these species is highest at this time (data not shown), and, therefore, the high water content ensures dilution. However, for day-flowering tobacco, the sugar concentration was also found to be lower during the dark period compared with the light period, even though the highest nectar volume is during the day, which contradicts the previous assumption. At night, phloem transport is reduced to approximately 40% of the daily rate (Riens et al., 1994), which means that less sucrose should arrive to the nectaries in darkness, and this could also be a reason for the observed fluctuations in the nectar sugar concentration. Therefore, it is generally easier for day-flowering plants to supply their nectar with nutrients for their pollinators, because they can process their metabolites directly from the phloem sap; in contrast, night-flowering plants, at least partially, have to store the metabolites (**Figure 8**). This finding corresponds to the differences in the starch content observed in the nectaries of day and night flowering species. In general, the night-flowering species had a higher starch content in the nectaries compared with the day-flowering species (**Figure 4**). Moreover, in night flowering species, the starch content decreased during the first half of the dark period, the time with high nectar production.

Starch accumulation may function as a form of sugar storage before anthesis (Weber et al., 1998), and starch degradation has been observed to occur before flower opening to provide additional sugar (Nepi et al., 1996; Horner et al., 2007; Ren et al., 2007b). In potato tubers (*Solanum tuberosum*), starch breakdown is triggered by decreased sucrose content (Hajirezaei et al., 2003). In this study, no overall correlation between starch and sucrose or hexoses in nectaries was observed, but in the case of nocturnal species, where there is a severe decrease of starch in the middle of the night, the sugar concentration was found to be significantly increased. Apart from that, there was no correlation between the starch content of the nectaries and leaves, thus the starch metabolism in the nectaries appears to function independently from the light-dependent starch metabolism of the plant.

Total nectary sugar concentration is highest at the time of flower opening, so sugar is likely provided for nectar production (**Figure 2**). There is a high correlation between the proportion of fructose and sucrose in nectaries and nectar. This suggests that the nectar sugar composition is already partly determined by the nectaries and is only partially adjusted during secretion. For glucose, this correlation is much lower. This phenomenon may be explained by the fact that some of the glucose is converted into starch and stored in the nectaries until it is used (Ren et al., 2007b).

### Modulation During Nectar Secretion

The sugar concentration in nectar was three to 10-fold higher than in the whole nectarial cells. An increase in concentration due to evaporation can be neglected because the analyzed species have very long and narrow flower tubes, which protect the nectar from evaporation (Plowright, 1987; Tiedge and Lohaus, 2017). This suggests that active sugar transport is involved in nectar secretion, perhaps through monosaccharide transporters (MSTs) and/or sucrose transporters (SUTs). A monosaccharide/proton symporter (AtSTP1), which only transports glucose but not fructose, has been found in *Arabidopsis* flowers (Sherson et al., 2003). SUTs have already been found in tobacco, as well, e.g., NtSUT3 in tobacco pollen (Lemoine et al., 1999), but, so far, their occurrence and function in flowers and nectaries is not completely understood.

A class of transporters that are clearly involved in nectar secretion are so-called SWEET sucrose transporters. In *Arabidopsis* and *Nicotiana*, SWEET9 functions as a facilitated diffusion transporter for sucrose (Lin et al., 2014), and there is evidence that this transporter is more responsible for sucrose efflux from nectarial cells than for sucrose uptake. As previously mentioned, the sugar concentration in nectar relative to nectary cells was three to fivefold higher in diurnal species and eight to 10-fold higher in nocturnal species. Unfortunately, until now, nothing has been reported about the subcellular distribution of sugars in the parenchyma cells of nectaries. Assuming that the subcellular distribution of sucrose in nectarial cells is similar to the distribution in leaves (up to 50% sucrose in the cytosol; Nadwodnik and Lohaus, 2008) and the cytosolic compartment comprises about 20% of the nectarial cells (Wist and Davis, 2006; Gaffal et al., 2007), the sucrose concentration in nectarial cells can be extrapolated (**Figures 2, 3**). In day-flowering species, the maximal sucrose concentration in the cytosol of nectarial cells was approximately 300–400 mM, and the corresponding concentration in nectar was approximately 100–300 mM. Similar results were obtained for *N. attenuata*. Therefore, it is possible that facilitated diffusion transporters for sucrose mediate sucrose efflux from nectarial cells (**Figure 8**). In night-flowering species, the calculated concentration of sucrose in the cytosol of nectarial cells was approximately 200 mM, whereas the corresponding concentration in nectar was approximately 300–700 mM. In the latter case, facilitated diffusion of sucrose from the nectarial cells into the nectar is not possible. However, this does not exclude the possibility that different cell types in the nectaries contain different sugar concentrations and that facilitated diffusion of sugars occurs only in certain nectarial cells, whereas in other cells active sugar secretion may occur. This finding is in line with findings in *Arabidopsis*, where SWEET9 was localized at the basal part of the nectaries (Lin et al., 2014), and the conclusions drawn from other research in this area, which propose a division of nectary parenchyma into functional sub-domains (Roy et al., 2017).

Besides sugar transporters, invertases also appear to be part of the nectar metabolism. For this work, the expression of the CW-INV was investigated exclusively in nectary tissue. Not much is known about the regulation of CW-INV expression in nectaries, but this enzyme has already been studied in other plant organs. Invertase expression is regulated by multiple factors, for example, by carbohydrates (Koch, 1996), phytohormones (Wu et al., 1993), biotic and abiotic stress-related stimuli (Roitsch et al., 2003), and proteinaceous inhibitors (Krausgrill et al., 1996). So far, it has rarely been examined how nectar-related invertase expression in nectaries is regulated. The invertase found in *N. attenuata* is highly upregulated in parts of early corollas, such as nectaries, ovaries and anthers. When the flowering continues to ripen, the invertase expression decreases (*Na*DH; Brockmöller et al., 2017). Most nectar is produced during early flowering, while older flowers sometimes have no nectar at all. This fact also suggests that invertase plays a role in the production of nectar. For other *Nicotiana* species, no organ-specific expression data about CW-INVs are available yet. Sturm and Chrispeels (1990) found that carrot cell suspension cultures grown on either glucose, fructose, or sucrose have similar β-fructofuranosidase mRNA content, with slightly higher levels of mRNA in cells grown on glucose (Roitsch et al., 1995). In contrast, the expression of different β-fructofuranosidase genes can be repressed by glucose (Kunst et al., 1974; Sarokin and Carlson, 1984; Martin et al., 1987). For tobacco, this phenomenon may only be applicable for *N. benthamiana*, where there is a strong negative correlation between the nectar sugars in general and invertase expression levels. Furthermore, high expression levels resulting in high CW-INV activity would have been expected. This seems to be true especially for species with high sucrose content in nectar (*N. sylvestris* and *N. tabacum*). Nevertheless, post-transcriptional processes seem to be taking place, which prevent the entire transcript from being converted into active protein.

The activity of CW-INV in the nectaries of different *Nicotiana* species (0.003–0.06 U mg^-1^ FW; **Figure 5**) was similar to the activity of CW-INV measured in other hexose-rich tissues of different plant species (Weschke et al., 2003). Moreover, an increased invertase activity would be expected in plants with a high hexose concentration in the nectar (Ruhlmann et al., 2010). However, for *tobacco*, this assumption is not confirmed by the data, regardless of whether the species is hexose-rich or not. The same applies to changes in the vacuolar invertase activities (Supplementary Figure 4A). In day-flowering species the vacuolar invertase activity was slightly higher in the light period and in night-flowering species in the dark period, regardless of whether the species is hexose-rich or not. Furthermore, due to the low activity, the neutral invertase seems to have only a relatively small influence on the hexose production. There might be other mechanisms that play a role in the sugar composition, for example, the *in planta* regulation of the sucrose cleavage enzymes. In addition to sucrose cleaving enzymes, sugar synthesis enzymes could also be involved in nectar production. It has been shown that sucrose phosphate synthase is highly expressed in some nectaries and that its expression can be essential for nectar production (Lin et al., 2014).

For all five *Nicotiana* species, the sucrose proportion of the total sugar concentration was always lower in the nectar compared with the nectaries (**Figure 3**), perhaps due to the extracellular hydrolysis of sucrose by CW-INVs. Differences in the sucrose-to-hexose-ratio between the nectaries and nectar were more pronounced in diurnal species compared with nocturnal species (Δ 0.37–0.48 vs. Δ 0.05–0.15). Therefore, the cleavage of sucrose during secretion must be stronger in diurnal species (**Figure 8**). Due to the differences in sugar composition between nectaries and nectar, especially in day-flowering species, it can be assumed that the sugar composition is at least partly modified during secretion, either by the selective transport of sugars and/or the activities of sugar cleavage enzymes, like CW-INVs.

### Post-secretory Modifications

No changes in nectar sugar concentration were observed after secretion in the tobacco species analyzed in this study and no invertase activity was detectable in nectar. In acacia, a significant post-secretional modification of extrafloral nectar by invertase has been demonstrated (Heil et al., 2005). Invertase activity in the nectar was also measured in *Cucurbita pepo*, but it was too low to significantly change the sugar profile (Nepi et al., 2012). Although other sugar-cleaving enzymes, such as glucosidase, have been identified in the nectar of *N. attenuata*, no invertases have been found in the tobacco nectar so far (Seo et al., 2013). This means that the nectar sugar composition must be already determined during the final stage of secretion, rather than undergoing post-secretory modification.

## Conclusion

Nectar sugar composition must be determined by metabolic processes in nectaries as well as during secretion (**Figure 8**). Sucrose is transported to the nectaries via the phloem. Within the nectaries, sucrose is hydrolyzed into hexoses, and a portion of the sugars is transiently stored as starch until anthesis, especially in night-flowering species. At anthesis, starch is converted into sucrose and hexoses. Sugars are exported out of the nectarial cells, likely by facilitated diffusion transporters (day-flowering species) or active transporters (night-flowering species). In the nectary tissue as well as during nectar secretion, some of the sucrose is hydrolyzed into glucose and fructose by the activity of CW-INVs, which explains the higher proportion of hexoses in nectar in comparison to nectaries. Sucrose cleavage is likely higher pre-secretional in night-flowering (possibly by vacuolar invertases) and during secretion in day-flowering species. Furthermore, post-secretional modification of the sugar composition in nectar is not probable. However, CW-INV alone cannot be responsible for the differences in hexoses concentration, and, therefore, other enzymes seem to play important roles in determining the nectar sugar composition.

## Author Contributions

KT and GL contributed conception and design of the study and wrote the manuscript. KT performed the statistical analysis.

## Conflict of Interest Statement

The authors declare that the research was conducted in the absence of any commercial or financial relationships that could be construed as a potential conflict of interest.
