# Supplementary material for: Nectar Sugar Modulation and Cell Wall Invertases in the Nectaries of Day- and Night- Flowering Nicotiana
Source: Front Plant Sci. 2018 May 9;9:622. doi: 10.3389/fpls.2018.00622 (PMC5954170; doi:10.3389/fpls.2018.00622)
Supplement: Supplementary file 1 [file Table_1.PDF]

Supplementary Material

Nectar Sugar Modulation and Cell Wall Invertases in Nectaries of day- and night- flowering *Nicotiana*

Kira Tiedge, Gertrud Lohaus*

Supplementary Table S 1: List of primers used in this paper

Species	Target gene	Primer	Sequence (5' - 3')
N. africana	CW-INV	forward	GTCCAAAACTACGCGATACC
		reverse	GATTTCTCACACTCCCAACC
	Actin	forward	GACTATGAGCAGGAACTTGAGA
		reverse	TGAACCACCACTAAGAACAATG
	Elongation factor 1α	forward	GGCCCAACACTTCTTGATGCTC
		reverse	CCAACATTGTCACCAGGAAGT
N. attenuata	CW-INV	forward	GAACTCCTTCCCTTCAGC
		reverse	GTTTGACACATTCCCGAC
	Actin	forward	CCCTCCCACATGCTATTCT
		reverse	TCCTGTTCATAGTCGAGAGC
	Ubiquitin-conjugating enzyme E2	forward	CTGACTTCAATCCAGTCTTTGCTC
		reverse	GTAACAGATTAAGAGTGCGGG
N. benthamiana	CW-INV	forward	GGCAATGATGAAGCACGATG
		reverse	ACTCCGGGATTGAATCAGG
	Actin	forward	TGTCCTGAGGTCCTTTTCC
		reverse	ATTCTATCAGCGATACCCGG
	Elongation factor 1α	forward	GGCCCAACACTTCTTGATGCTC
		reverse	CCAACATTGTCACCAGGAAGT
N. sylvestris	CW-INV	forward	TGATTCTTCACACTCCCAACCA
		reverse	CACAAGTCCAAAACTACGCGA
	Actin	forward	GACTATGAGCAGGAACTTGAGA
		reverse	TGAACCACCACTAAGAACAATG
	Elongation factor 1α	forward	GGCCCAACACTTCTTGATGCTC
		reverse	CCAACATTGTCACCAGGAAGT
N. tabacum	CW-INV	forward	CTGCTATGTGCTGATGGATG
		reverse	CAGGCGCTTCACTATTCTC
	Actin	forward	CCCTCCCACATGCTATTCT
		reverse	TCCTGTTCATAGTCGAGAGC
	Elongation factor 1α	forward	CTGCTTATTGACTCCACCAC
		reverse	CACCTTCCAAACCAGAGATG
