# Supplementary material for: Nectar Sugar Modulation and Cell Wall Invertases in the Nectaries of Day- and Night- Flowering Nicotiana
Source: Front Plant Sci. 2018 May 9;9:622. doi: 10.3389/fpls.2018.00622 (PMC5954170; doi:10.3389/fpls.2018.00622)
Supplement: Supplementary file 3 [file Image_2.PDF]

Supplementary Material

Nectar Sugar Modulation and Cell Wall Invertases in Nectaries of day- and night- flowering *Nicotiana*

Kira Tiedge, Gertrud Lohaus*

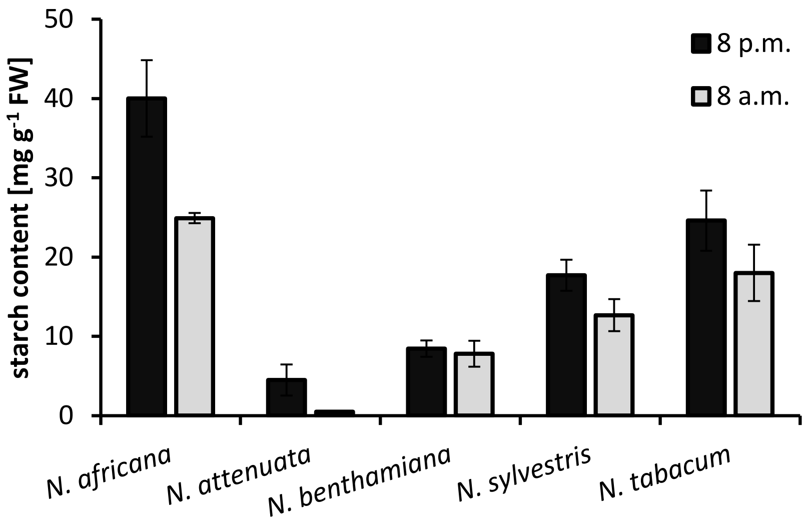

Supplementary Figure 2. Starch content of tobacco leaves, which have been collected both at the end of the light period (8 p.m.) and at the end of the dark period (8 a.m.); shown are mean values (*n* = 3) ± SD
