# Supplementary material for: Nectar Sugar Modulation and Cell Wall Invertases in the Nectaries of Day- and Night- Flowering Nicotiana
Source: Front Plant Sci. 2018 May 9;9:622. doi: 10.3389/fpls.2018.00622 (PMC5954170; doi:10.3389/fpls.2018.00622)
Supplement: Supplementary file 5 [file Image_4.PDF]

Supplementary Material

Nectar Sugar Modulation and Cell Wall Invertases in Nectaries of day- and night- flowering *Nicotiana*

Kira Tiedge, Gertrud Lohaus*

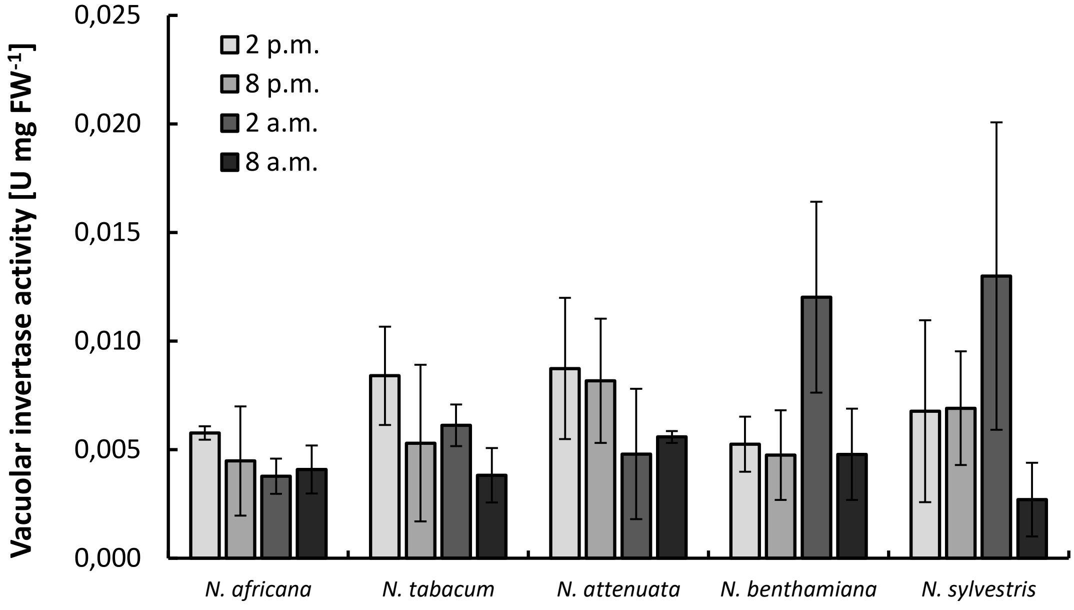

Supplementary Figure 4A. Vacuolar invertase activity in nectary tissue. Mean values from all measurements taken at one time point (*n* = 3) and the respective SD were plotted.

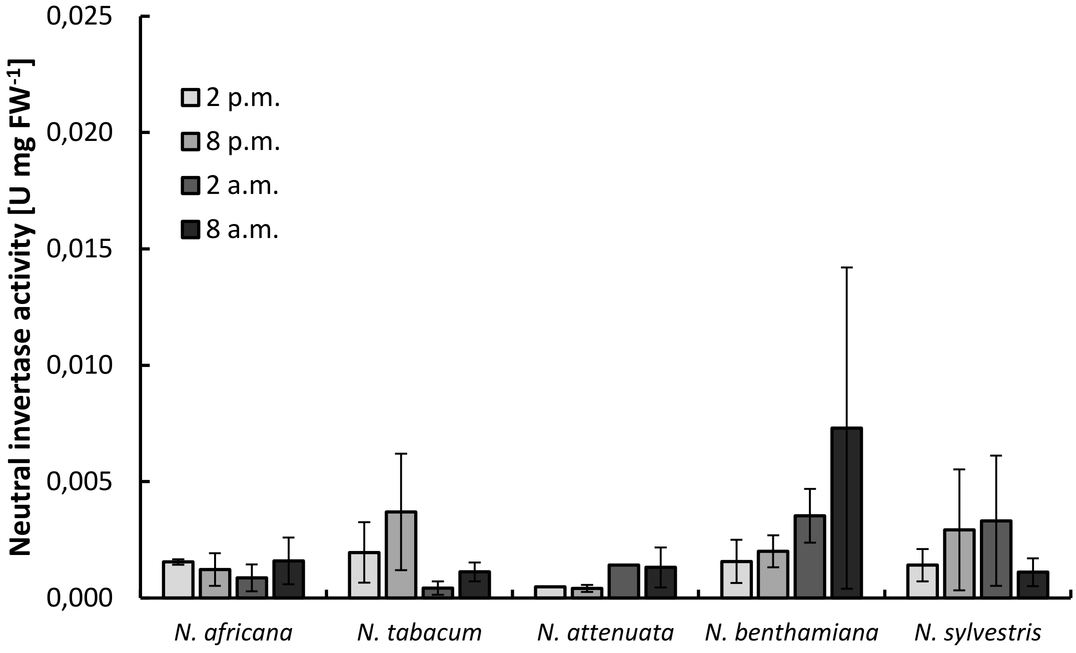

Supplementary Figure 4B. Neutral invertase activity in nectary tissue. Mean values from all measurements taken at one time point (n = 3) and the respective SD were plotted.
